# Recent advancements in nanomaterial-based biosensors for diagnosis of breast cancer: a comprehensive review

**DOI:** 10.1186/s12935-025-03663-8

**Published:** 2025-02-18

**Authors:** Yalda Yazdani, Fereshtehsadat Jalali, Habib Tahmasbi, Mitra Akbari, Neda Talebi, Seyed Abbas Shahrtash, Ahmad Mobed, Mahsa Alem, Farhood Ghazi, Mehdi Dadashpour

**Affiliations:** 1 Immunology Research Center, Tabriz University of Medical Sciences, Tabriz, Iran; 2 Department of Obstetrics and Gynecology, Faculty of Medicine, Shahid Beheshti University of Medical Sciences, Tehran, Iran; 3 Department of Microbiology and Biotechnology, Faculty of Biotechnology, Bangalore University, Bangalore, India; 4 Eye Research Center, Eye Department, Amiralmomenin Hospital, School of Medicine, Guilan University of Medical Science, Rasht, Iran; 5 Neuromuscular Rehabilitation Research Center, Semnan University of Medical Sciences, Semnan, Iran; 6 Department of Pharmaceutical Engineering, University of Tehran, Tehran, Iran; 7 Social Determinants of Health Research Center, Tabriz University of Medical Sciences, Tabriz, Iran; 8 Department of Microbiology, Faculty of Veterinary Medicine, Urmia University, Urmia, Iran; 9 Clinical Research Development Unit of Tabriz Valiasr Hospital, Tabriz University of Medical Sciences, Tabriz, Iran; 10 Cancer Research Center, Semnan University of Medical Sciences, Semnan, Iran; 11 Department of Medical Biotechnology, Faculty of Medicine, Semnan University of Medical Sciences, Semnan, Iran; 12 Department of Medical Biotechnology, Semnan University of Medical Sciences, Semnan, Iran

**Keywords:** Breast cancer (BC), BRCA-1,2, Nanomedicine, Nanotechnology, Biosensors

## Abstract

Researchers have found that mutations in the BRCA gene associated with breast cancer have a 40–50% chance of being associated with high risk for hereditary breast cancer (BC). Therefore, detecting BRCA1 is crucial for genetic analysis, early detection, and clinical treatment of BC. Traditional detection methods for BRCA1 include high-performance liquid chromatography (HPLC), single-strand conformation polymorphism assays (SSCP), PCR, real-time PCR, and DNA sequencing. However, these methods are limited by cost, analysis time, and complexity. Therefore, it is necessary to develop an ultrasensitive, fast, low-cost, simple method for BRCA1 detection. In recent years, various BC biosensing strategies have been investigated, including optical, electrical, electrochemical, and mechanical biosensing. In particular, the high sensitivity and short detection times of electrochemical biosensors make them suitable for recognizing BC biomarkers. Additionally, the sensitivity of electrochemical biosensors can be increased by incorporating nanomaterials. In this regard, the main focus of the present study is the introduction of common methods for diagnosing the BRCA-1/2 genes. In addition to introducing biosensors as an efficient tool, it also discusses the latest and most significant biosensors developed for detecting the BRCA gene.

## Introduction

Hereditary breast and ovarian cancers (HBOCs) associated with BRCA1 and BRCA2 include male and female breast cancer (BC), ovarian cancer (as well as primary peritoneal cancers and fallopian tube cancers), and, to a lesser extent, prostate cancer [1, 2]. In 2020, there were approximately 2.3 million new breast cancer cases and 685,000 breast cancer-related deaths worldwide [3–5]. The frequency and mortality rates vary among nations, with the age-standardized incidence ranging from a high of 112.3 per 100,000 people in Belgium to a low of 35.8 per 100,000 people in Iran. Similarly, the age-standardized mortality rates range from a high of 41.0 per 100,000 people in Fiji to a low of 6.4 per 100,000 people in South Korea [3]. The BRCA1 gene is responsible for producing proteins that aid in the repair of damaged DNA. Detecting BRCA1 and its mutations with high sensitivity is crucial for enabling early diagnosis, as harmful mutations in this gene significantly increase the risk of developing ovarian or breast cancer [6, 7]. Individuals with predominantly BRCA2 pathogenic variants are characterized by an increased risk of breast cancer, pancreatic cancer, and ovarian cancer. Estimates of the risk of malignancies vary widely depending on the context in which they are derived [1, 2]. The lifetime risk of breast cancer for women who inherit damaging germline BRCA1 or BRCA2 mutations is substantial; by the age of 80, the anticipated rates are 72% and 69%, respectively [8]. After being diagnosed with invasive breast cancer, these women are at an increased risk of developing secondary ipsilateral breast cancer [8, 9]. Mutations in the BRCA1 and BRCA2 genes significantly increase the lifetime risk of progression to ovarian and breast cancer, and these mutations are frequently found in hereditary breast and ovarian cancer cases [10]. Additionally, altered and dysregulated BRCA1 and BRCA2 protein expression enhances the risk of sporadic breast cancer. Both genes play critical roles in DNA repair and transcriptional regulation in response to DNA damage [10, 11]. Research indicates that the most well-known function of BRCA1 is in the DNA repair pathway, and some clinically observed missense mutations throughout the BRCA1 gene are functional in the DNA double-strand break (DSB) repair assay [12]. These findings demonstrate an association between the efficient repair of DSBs by BRCA1 and its tumor-suppressive activity [12, 13]. Carriers of BRCA1 mutations are generally sensitive to platinum-based therapies, while tumor cells expressing high levels of BRCA1 are resistant to ionizing radiation (IR) and chemotherapeutic agents [12, 13]. Breast cancer monitoring in BRCA carriers includes monthly self-examinations, 1–2 annual clinical breast examinations, and mammograms and magnetic resonance imaging (MRI) starting at ages 25–30 [14]. Mutations in the BRCA1 and BRCA2 genes are among the primary causes of breast and ovarian cancer. Therefore, rapid and specific detection of these genes can play a vital role in diagnosing and monitoring cancer. In this regard, the present study addresses the diagnostic methods for BRCA1 and BRCA2, which include routine techniques such as PCR, SSCP, MLPA, and Next Generation Sequencing (NGS). Additionally, nanotechnology-based methods, including biosensors, are highlighted and discussed in this study.

### Overall challenges in breast cancer recognition and management

The various molecular types of breast cancer (BC) dictate the treatment approach, including targeted therapies such as endocrine therapy for HR + BC and anti-HER2 therapy for HER2 + BC [15]. These treatments are not only regarded as safe but also effective in many instances [15]. However, the development of advanced and modern treatments is essential, as breast cancer is a complex disease and not all patients can be effectively treated with conventional methods alone [15, 16]. For example, one of the main challenges in treating breast cancer is finding effective advanced therapies for patients with triple-negative breast cancer (TNBC), as standard treatments for this specific type have proven ineffective and have yielded the poorest outcomes [16, 17]. Another significant challenge in the treatment of breast cancer is treatment resistance, which is a common issue in the management of endocrine abnormalities, anti-HER2 therapy, and chemotherapy [18, 19]. To develop new treatments for breast cancer, it is essential to understand the mechanisms underlying drug resistance. For instance, the mTOR/PI3K/Akt pathway plays a significant role in drug resistance across all molecular subtypes of breast cancer, making the development of inhibitors that specifically target these pathways highly promising and valuable. Targeted therapies based on nanomaterials are becoming increasingly important and are now considered a viable option for many incurable diseases. Nevertheless, progress in treating triple-negative breast cancer (TNBC) remains a significant challenge for the oncology community. In clinical practice, standard chemotherapy regimens, including anthracyclines and taxanes, are still commonly used. Meanwhile, the use of platinum-based chemotherapy in TNBC is gaining traction, particularly among patients with BRCA-associated mutations [18, 19]. Conversely, clinical research in the neoadjuvant setting has concentrated on two main areas: the development of immunotherapy drugs and innovative targeted therapies. Given the significance of heterogeneity in triple-negative breast cancer (TNBC), conducting studies in selected patient populations presents a greater challenge [19]. The most promising new approaches include immunotherapy with checkpoint inhibitors, poly (ADP-ribose) polymerase (PARP) inhibitors, and androgen receptor (AR) inhibitors. Additionally, active research is ongoing to identify further specific targets in TNBC [19].

### The potential role of nanomedicine in breast cancer treatment to overcome the obstacles of current therapeutic approaches

Nanotechnology has emerged as one of the most significant fields in the past 20 years for developing novel treatment approaches for breast cancer [20–22]. Key advantages of nanomaterial-based drugs include reduced toxicity and the ability to overcome drug resistance, particularly in chemotherapy [23, 24]. The use of nanomedicines has proven highly effective in treating various types of breast cancer tumors. Different types of nanoparticles, such as liposomes, polymer nanoparticles, polymer micelles, dendrimers, and carbon nanotubes, have been explored and utilized for targeted drug delivery [24]. Lipid-based nanocarriers have shown some of the most promising results among all types of nanocarriers, particularly when combined with synthetic chemotherapeutic agents and antitumor bioactive molecules [25]. Nano-material-based approaches can overcome the problems of drug resistance and cancer stem cells [25]. In the coming decades, researchers should focus on lipid-based nanocarriers using a combinatorial strategy to enhance the lives of breast cancer patients [25, 26]. Like all diagnostic and therapeutic methods, those based on nanomaterials have certain limitations, although these are generally less significant compared to older, commonly used techniques. For instance, many nanocarriers can be toxic to healthy tissues and may stimulate the immune system [27, 28]. However, targeted therapy can be very expensive in some cases. Therefore, future research should focus on overcoming these limitations, particularly by reducing the toxicity of nanomaterials not only to the body but also to the natural environment [26, 27].

Breast cancer (BC) is the second most commonly reported cancer in women, with a high mortality rate, resulting in millions of cancer-related deaths each year. Early detection of BC is crucial in the fight to discover, develop, and optimize diagnostic biomarkers that can improve prognosis and treatment outcomes [29–31]. BC-related biomarkers include macromolecules such as proteins, nucleic acids (DNA/RNA), and intact cells. Advances in molecular technology have identified various biomarkers specifically studied for drug resistance, as well as for diagnostic, prognostic, and therapeutic purposes. Recognizing these biomarkers could help address the problem of drug resistance, which is a significant obstacle in BC treatment. Several studies indicate that gene-based biomarkers play a critical role in BC diagnosis, treatment, and screening [29–31]. Accordingly, BC-related genes were discussed in the next section.

## Genetics and risk of breast cancer

**Fig. 1:**
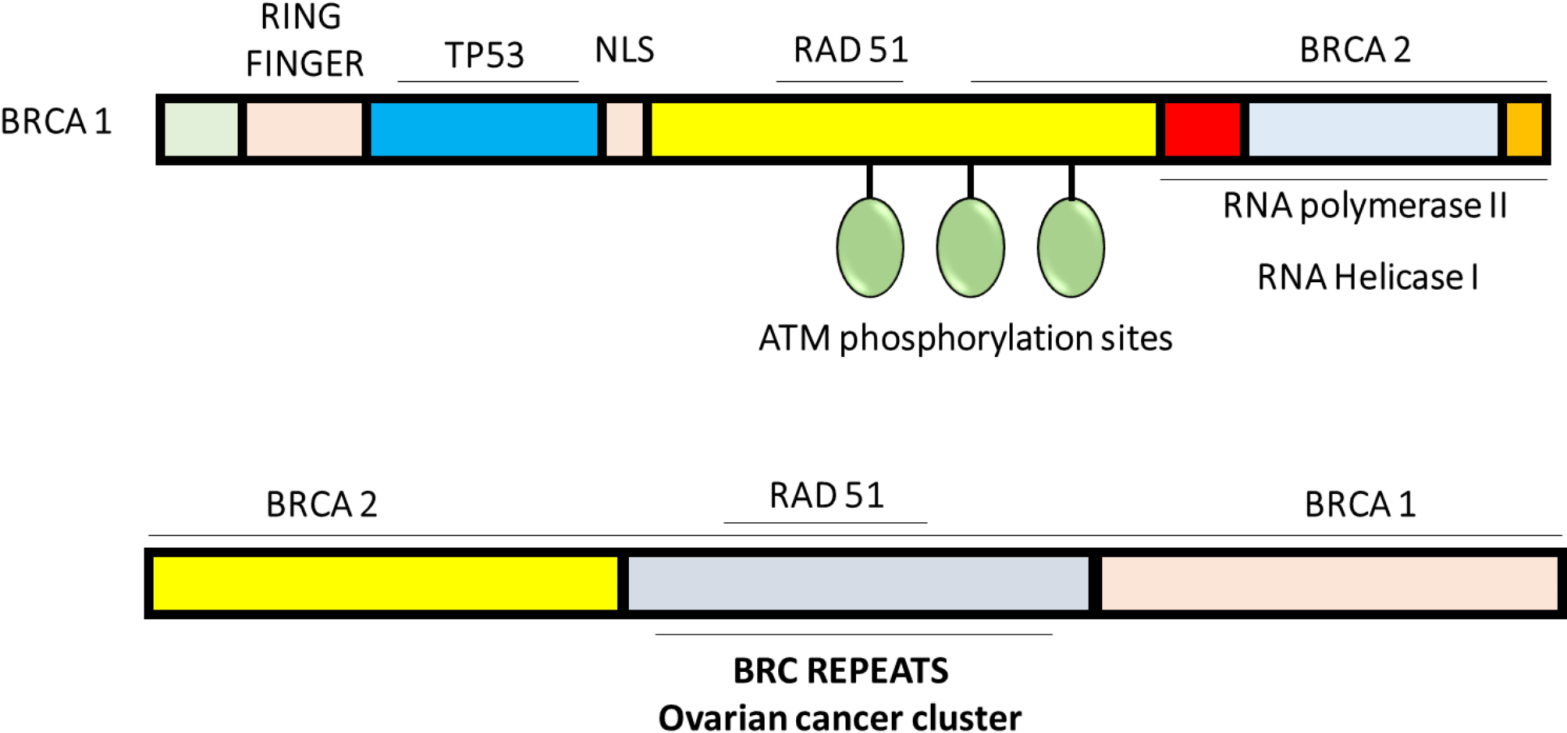
Schematic representation of BRCA1 and BRCA2 genes [34]

The term ‘BRCA’ is an abbreviation for ‘breast cancer gene.’ BRCA1 and BRCA2 are two distinct genes that influence the likelihood of developing breast cancer [32, 33]. A demographic illustration of the BRCA genes is presented in Fig. 1.

Everyone has both BRCA1 and BRCA2 genes. Contrary to what their name suggests, the BRCA genes do not directly cause breast cancer [32, 33]. However, certain hereditary mutations in these genes are associated with an increased risk of developing cancer, primarily linked to the tumor suppressor functions of BRCA1 and BRCA2 [35, 36]. Specifically, the BRCA1 gene encodes a protein consisting of 1,863 amino acids that is linked to chromosomal stability. Mutations in this gene are closely associated with an early onset of familial breast cancer syndrome and play a crucial role in regulating cell cycle checkpoints and cell division[37, 38]. Mutations in the BRCA1 and BRCA2 genes are linked to an increased risk of breast cancer and account for approximately 21–40% of hereditary breast cancer cases. Additionally, BRCA1 protein expression has been reported to be decreased in 30% of sporadic breast cancer cases. The degree of BRCA1 protein reduction correlates with the severity of the breast cancer and is inversely related to the expression of BRCA2, which can also serve as a tool for treating sporadic breast cancer [30]. Furthermore, BRCA2 can be utilized as both a prognostic and a screening biomarker for breast cancer.

### Conventional and molecular-based methods in the detection of breast cancer genes

BRCA1 germline mutations, primarily point mutations and other minor alterations, are responsible for the majority of hereditary breast cancer cases [39]. However, the observed frequency of BRCA1 changes is lower than what linkage analysis would predict. Various engineering approaches have identified multiple significant BRCA1 rearrangements in certain families. A gene-dose assay method was developed for real-time quantitative analysis of breast cancer, but it failed to detect point mutations in BRCA1 or BRCA2. This method quantifies the number of copies of each BRCA1 exon, allowing for the easy detection of one, two, or more copies of a specific BRCA1 target exon [40]. The frequency of BRCA1 gene mutations was analyzed using allele-specific real-time PCR on appropriately pooled genomic DNA samples [41]. A real-time quantitative RT-PCR method (QRT-PCR) was proven for the sensitive determination of BRCA1 mRNA [42]. Single-stranded conformation polymorphism (SSCP) analysis is a widely used screening method that enables the identification of various genomic variants across a large number of samples and a broad range of organisms, from microorganisms to humans [43]. Mutations in BRCA1 were screened using SSCP for shorter exons, while direct sequencing was employed for longer exons [44]. The results indicate that SSCP analysis may serve as an ideal platform for identifying both somatic and hereditary germline mutations that contribute to cancer. This approach provides a foundation for DNA-based cancer classification, aids in identifying genes that need regulation, and advances our understanding of cancer based on the biochemical functions of allelic variants that can hinder cancer progression and enhance the consideration of potential therapeutic targets [45]. MLPA is a method that expands a pool of custom probes into specific genomic regions and is used to detect specific small chromosomal abnormalities, as well as single or partial gene deletions [46, 47]. With the power of next-generation sequencing (NGS) analysis, it is possible to analyze genes related to genetic susceptibility to breast cancer and to study genetic etiology more thoroughly. The results indicate that NGS is a cost-effective gene panel approach. It is recommended to use the gene panel as a first-line genetic test for hereditary breast cancer and to consider MLPA analysis of the BRCA1/2 genes as a complement to NGS analysis [48]. BRCA1/BRCA2 mutations in the ovarian cancer population were detected using both MLPA and NGS analysis. The overall proportions of BRCA mutations in both somatic and germline cases are consistent with global data and are more likely to be diagnosed by MLPA, which is limited by blood samples with low germline large genomic rearrangement (LGR) rates. NGS is becoming the method of choice for targeting both small mutations in the BRCA genes and large genomic rearrangements (LGR) [49] (see Tables 1 and 2).

**Table 1:** The highlights of conventional methods in the detection of BRCA

BRCA-1,2	Method	Comments and significances	Ref
BRCA-1	Real-time PCR-based gene dosage	This method should be seen as a powerful diagnostic tool for both ovarian and BC susceptibility in clinical and research genetic studies.	[39]
BRCA-1	Allele-specific real-time PCR of pooled genomic DNA samples	The developed system was an effective tool for early cancer detection among mutation carriers.	[41]
BRCA-1	Quantitative RT-PCR method (QRT-PCR)	The established BRCA1 QRT-PCR method was ultra-sensitive, quantitative, and specific. The planned system was rapid, automatic, and cost-effective and can be used to study BRCA1 expression in a wide range of clinical samples.	[50]
BRCA-1	SSCP	Five mutations were determined by developed approach.	[44]
BRCA-1	SSCP	The developed method was able to screen of the BRCA1 gene in the population.	[45]
BRCA1,2	NGS	Findings present that NGS was the low-cost and effective of the BRCA1,2 gene panel approach.	[48]
BRCA-1,2	MLPA, NGS	NGS is becoming the method of choice targeting both small mutations in the BRCA gene and LGR.	[49]

According to studies, traditional breast cancer risk (BCR) diagnostic techniques are useful and significant, but they have drawbacks, including complexity and high costs. To address these limitations, various alternative methods are being developed. Among these, nanotechnology-based methods, such as biosensors, have garnered significant interest from researchers over the past two decades. The next section of the article will cover biosensor technology, its advantages, and analytical properties, along with a comprehensive introduction to the latest biosensors for detecting breast cancer genes as critical biomarkers for breast cancer.

## Biosensors methodology, construction, and classification

Currently, most biomarker testing is performed in dedicated centralized laboratories using large automated analyzers, which increases wait times and costs. There is a growing demand for smaller, faster, and more affordable devices to replace these time-consuming laboratory analyses and provide analytical results at the bedside (point-of-care diagnostics) [51, 52]. Innovative strategies based on biosensors can enable reliable testing of biomarkers in decentralized environments; however, there are still challenges and limitations that need to be addressed in the design and application of biosensors for the appropriate interpretation and quantification of identified biomarkers [51, 52]. The development of biosensors is one of the most promising approaches to addressing the need for highly sensitive, rapid, and economical analytical methods in diagnostics. Biosensors and point-of-care (POC) devices have the potential to transform healthcare, as biosensor technology can be utilized in low-cost, disposable point-of-care devices [53, 54]. Alternatively, biosensors can facilitate continuous monitoring of embedded devices. However, measurements in biomedicine pose distinct challenges in terms of both implementation and interpretation [53, 54].

**Fig. 2:**
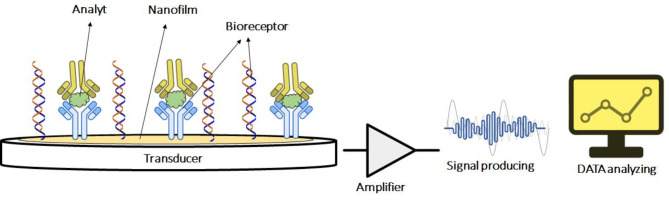
Schematic illustration of biosensors technology. The biosensor contains the following four structures: sensing elements (or receivers), an interface that provides a working environment for the biosensing elements, a transducer, a series of electronics including signal amplification, signal processing, and interface circuits for data analysis and processing

**Fig. 3:**
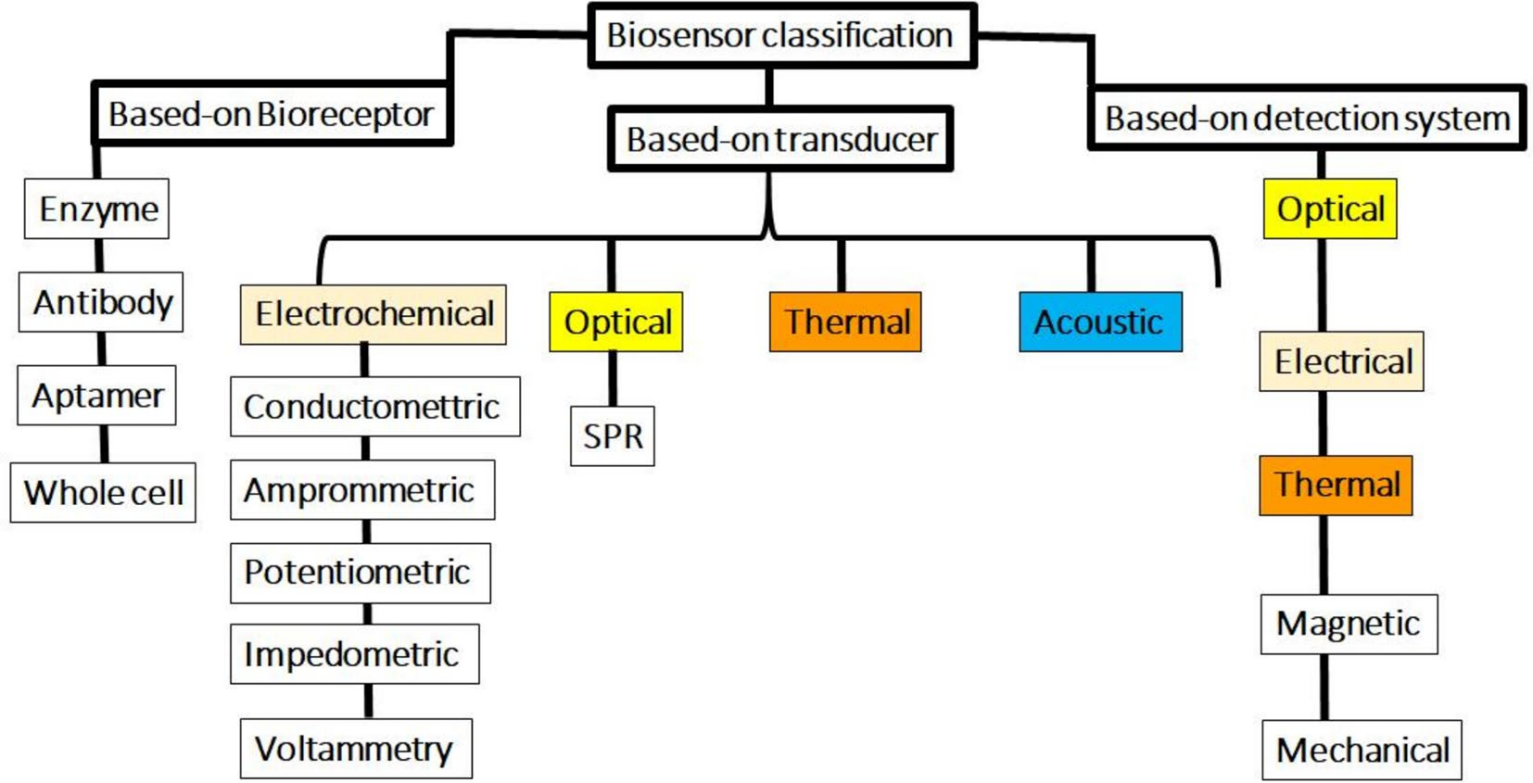
Biosensors classification, adapted from ref [64]

**Fig. 4:**
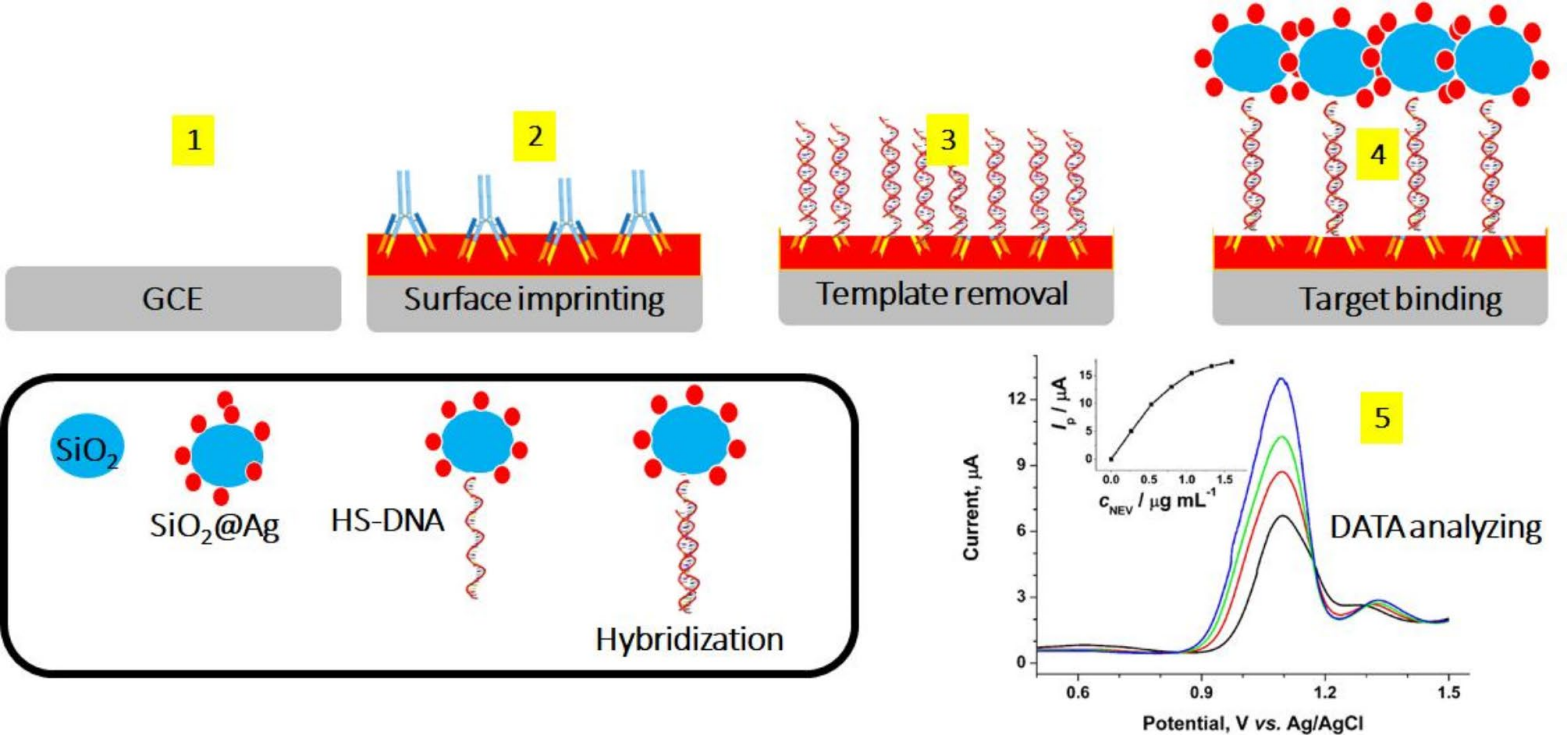
Schematic illustration of the MIP-based E-DNA biosensor. The surface printing is performed on the bare GCE electrode and the preparation of SiO2@Ag/DNA and the homogenous hybridization of DNA are performed in step 4. As shown, the target DNA is homogenously hybridized with SiO2@Ag-modified DNA and RhB-labeled DNA, forming a recognizable SiO2@Ag/dsDNA/RhB nanocomposite. Adapted from ref [68]

**Fig. 5:**
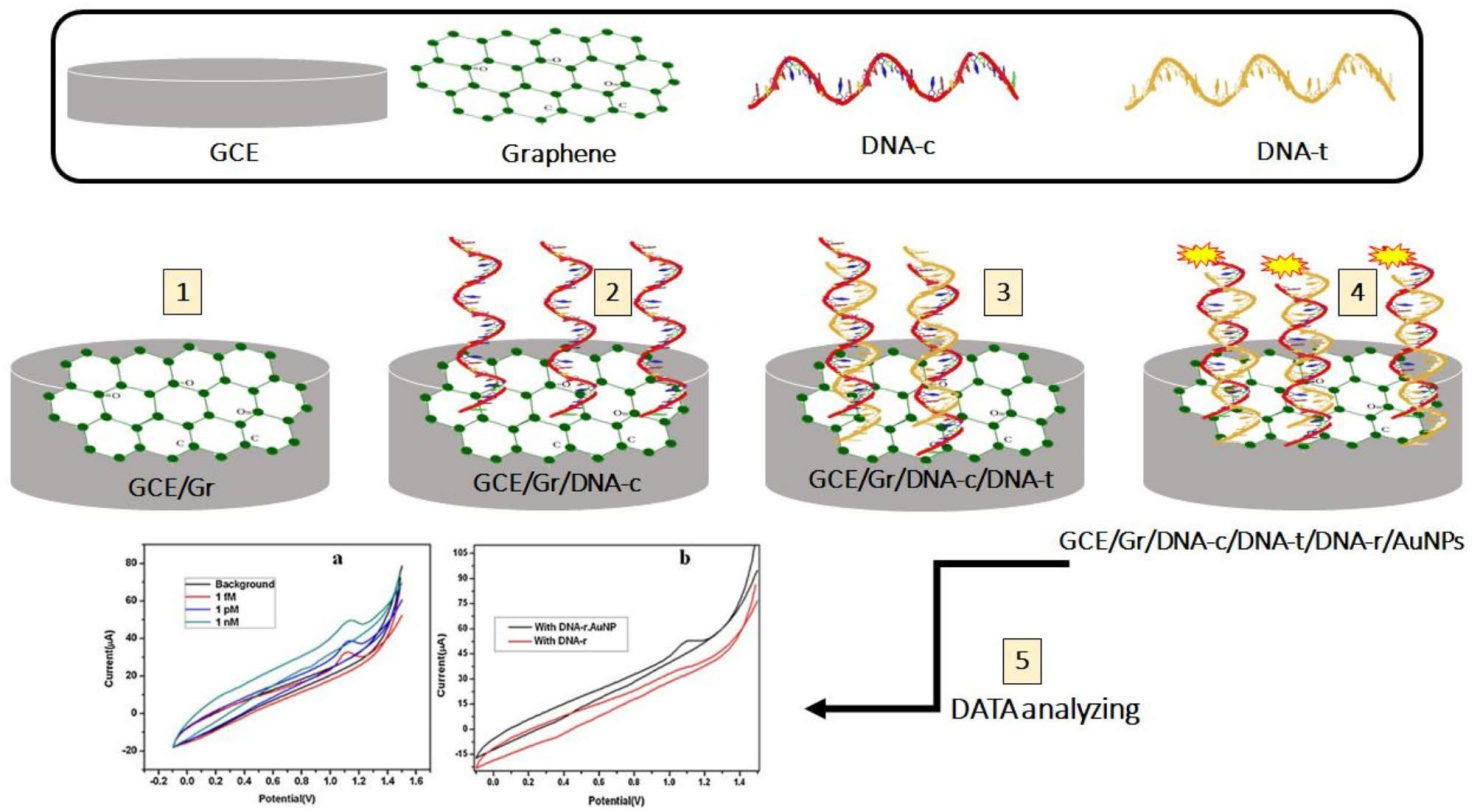
The representation of the various stages of sensor fabrication related to specific BRCA1 sequences of sensor fabrication. As presented in step 1, the bare GCE electrode was modified with the graphene, DNA hybridization occurred in steps 3 and 4, and data analysis was performed in step 5. Adapted from ref [72]

**Fig. 6:**
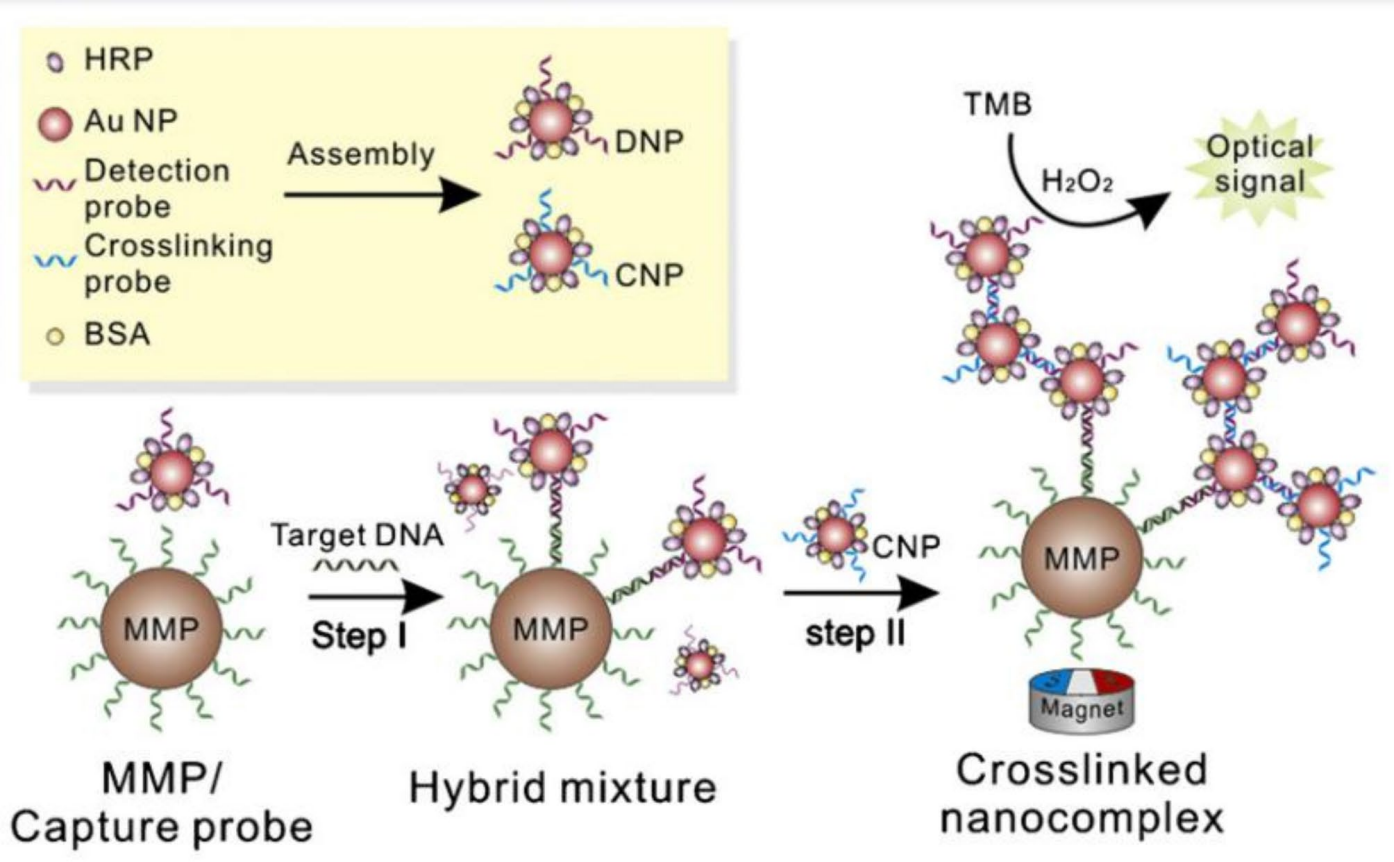
An assay for detecting an amplified BRCA-1 gene sandwich using multifunctional cross-linked Au aggregates and magnetic particles is described. As shown, probe collection on MMP takes place in step 1 and hybridization is performed in step 2. Accordingly, target DNA detection and optical detection take place after binding horizontal of the nanocomposite, with permission from ref [76]

Biosensors have been widely researched and developed as tools in the fields of medicine, environment, food, and pharmaceuticals [55, 56]. They are designed to generate digital electronic signals that are proportional to the concentration of a specific biochemical substance or a range of biochemical substances in the presence of various interfering species. These devices are referred to as “biosensors” because they utilize biological features such as detection and catalysis [57]. The typical architecture of a biosensor combines biological components with transducers. Amperometric enzyme biosensors, which consist of enzymes as biomaterials and electrodes as transducers, are among the most popular types of biosensors [57, 58]. The fact that the output signal is a current simplifies the design of the measurement circuit and enhances its sensitivity compared to biosensors that measure potential difference [57, 58] (see Figures 1, 2, 3, 4, 5 and 6).

Biosensors can be classified according to their physicochemical delivery method or the type of biosensor. Based on the probe, biosensors can be classified into electrochemical, optical, thermal, and piezoelectric biosensors [59, 60]. Electrochemical biosensors can be classified as amphoteric biosensors (measures the current generated during the oxidation or reduction of an electrochemical product or reagent), potentiometric biosensors (measures the potential of the biosensor electrode relative to the reference electrode), and biometric sensors (which measures the change in electrical conductivity due to a biochemical reaction) [59–61]. Electrochemical biosensors are the most studied biosensors because they have the advantages of low detection limit, specificity, simple structure, and ease of use. With recent advances in electronic instrumentation, these biosensors can be miniaturized as lab on chip devices for in vivo monitoring or as wearables for on-site monitoring [62, 63].

As shown in the figure, biosensors are categorized into three main group based-on biorecptor, transducer, and detection system The main components of biosensors are biological recognition elements and physical/chemical transducers. Due to these biorecognition mechanisms, biosensors have noteworthy advantages such as high selectivity, high sensitivity, and the capability for high-throughput processing. Therefore, the development of biosensors has accelerated and progressed significantly in recent years. The present review study continues to introduce and discuss the latest biosensors developed for the diagnosis of BRCA-1/2 genes associated with breast and ovarian cancer.

## Advanced biosensors platform for ultra-sensitive detection of BRCA

Electrochemical assays have the potential to provide a very inexpensive, automated, portable and sensitive method for the detection of a wide variety of disease biomarkers, minimizing non-specific adsorption and are important issue for applications in natural complex media [65, 66]. An electrochemical biosensor was developed and applied for sensitive and selective detection of the BRCA1. It is based on zwitterionic self-assembled peptide monolayer (SAM) support that acts as a low fouling substrate and is 19 mer BRCA1-related sequence specific. The created system performed as a sensitive label-free sensor approach for oligonucleotides and electrochemical impedance spectroscopy (EIS) [67].

An innovative electrochemical DNA (E-DNA) biosensing approach was aimed and used for the recognition of the BC susceptibility gene (BRCA-1). The planned system was based on gold nanoparticles-reduced graphene oxide (AuNPs-GO) modified glass carbon electrode (GCE) covered with the layer of molecularly imprinted polymers (MIPs) synthesized with methacrylic acid (MAA) as the monomer, rhodamine B (RhB) as template, and Nafion as additive [68].

A numerical illustration of a hybrid strategy was developed, along with a numerical study of graphene-coated fiber-optic surface plasmon resonance (SPR) biosensors for the recognition of BRCA-1 and BRCA-2 genetic mutations associated with breast cancer (BC). Specifically, two mutations were targeted: 916delTT in the BRCA1 gene and 6174delT in the BRCA2 gene. This method employed Attenuated Total Reflectance (ATR) technology to detect these single-point mutations in the BRCA1 and BRCA2 genes [69]. Smartphone-assisted colorimetric detection of the BRCA-1 gene based on catalytic hairpin assembly amplification and G-quadruplex DNAzyme was organized recently. The created colorimetric biosensor displays outstanding selectivity and suitable applicability for the detection of the BRCA-1 gene in human serum samples with acceptable recoveries linearity which might hold excessive application potential in genetic analysis and clinical diagnosis [70]. An ultrasensitive label-free electrochemical DNA (E-DNA) biosensor has been developed using a conducting polymer/reduced graphene oxide platform for the detection of the BRCA1 gene. In this platform, an electrochemical method was employed as a straightforward and efficient approach for the reduction of graphene oxide (GO), as well as for the electro-polymerization of pyrrole-3-carboxylic acid monomer [71]. Graph-based electrochemical DNA sensors have been developed to detect low levels of the BC-related BRCA1 gene. The DNA sensor employs a “sandwich” detection strategy in which the capture probe (DNAc) and reporter probe (DNAr) DNA hybridize to the target probe DNA (DNAt) in a sandwich preparation on a graphene-modified glassy carbon electrode (GCE). The DNAr was bound to AuNPs and the oxidation of the AuNPs was used for the electrochemical detection of DNAt [72].

A carbon nanotube-modified glassy carbon electrode (CNT/GCE) has been developed for the amplified label-free electrochemical detection of DNA hybridization, specifically targeting the BRCA-1 gene. The advantages of CNT/GCE electrodes are highlighted in comparison to conventional unmodified glassy carbon, carbon paste, and graphite pin electrodes. The enhanced amplification of the guanine signal was achieved in conjunction with label-free electrical detection of DNA hybridization [73]. In situ hybridization chain reaction (HCR)-mediated electrochemical biosensors, which are enzyme-free and conjugation-free, have been developed for the sensitive detection of the BRCA-1 gene in complex matrices. The HCR-based genosensor can directly detect low-frequency BRCA1 gene sequences in complex samples, including 50% human serum, with minimal interference. These advantages make customized HCR-based electrochemical genosensors highly attractive for genetic analysis and clinical diagnosis [74]. The monolithic silicon optocoupler was developed as an affordable device for the sensitive detection of the BRCA1 gene. In this optocoupler design, a system for real-time detection of oligonucleotides corresponding to both wild-type and mutant-type sequences was immobilized onto different optocouplers. The hybridization process with fluorescently labeled complementary or non-complementary sequences was monitored effectively [75]. A novel sandwich assay has been developed for the optical detection of DNA using agglomerates of cross-linked multi-component gold nanoparticles (AuNPs). In this project, the detection probes consist of DNA cross-linking multifunctional AuNP aggregates that integrate DNA recognition (detection probe), signal amplification (using the enzyme horseradish peroxidase), and non-specific blocking agents (such as bovine serum albumin, BSA). This assay is specifically designed to detect the BRCA1 gene associated with breast cancer (BC). The detection limit achieved is acceptable and shows significant improvement compared to results obtained from a single assay using labeled Au nanoparticles [76].

A label-free, target-recycling electrochemical impedance spectroscopy (EIS) genosensor has been developed for the detection of a model related to the BRCA-1 gene, achieving an acceptable LOD. This sensor can effectively distinguish between complementary DNA, single-base mismatch DNA, and non-complementary DNA. As a result, it shows promise for the early detection of BRCA-1 mutations. Furthermore, this strategy is anticipated to have significant potential for studying DNA hybridization and may offer advantages in the diagnosis of viruses and various diseases [77]. An electrochemical study was conducted to observe the hybridization of a specific sequence on BRCA1 DNA using a single-walled carbon nanotube (SWCNT)-based screen-printed graphite electrode (SPE). The developed system demonstrated high selectivity and sensitivity for the detection of BRCA1 DNA [78]. Research studies have explored low-cost electrochemical DNA sensors based on DNA tetrahedral structures. These sensors utilize gold nanoparticles (AuNPs) in conjunction with a tetrahedral DNA probe (TSP) and polyadenylation (polyA) for the ultrasensitive detection of BRCA1. Thiol-modified TSP serves as a scaffold on the surface of screen-printed AuNP electrodes. In this system, the captured TSP and reporter DNA hybridize with the target DNA (BRCA1) to form a typical sandwich assay [79]. An electrochemical DNA biosensor has been developed for the rapid detection of sequence-specific BRCA1 genes. This electrochemical nanosensor is based on a short oligonucleotide DNA probe that is immobilized on zinc oxide nanowires (ZnONWs). The ZnONWs are chemically synthesized on gold electrodes using hydrothermal technology [80]. An important technique has been developed to detect the 185delAG mutation in the BRCA1 gene, which is associated with a lifetime risk of ovarian cancer and hereditary breast cancer in women. The proposed method is based on the quenching and restoration of fluorescence of fluorescein dye (FAM)-labeled DNA in the presence of graphene oxide (GO), followed by the addition of complementary DNA (cDNA). Additionally, a ligase reaction is employed between a GO-bound DNA probe and FAM-labeled DNA at the 5’ end in the presence of cDNA [81]. Electrochemical nanobiosensors are employed to interact with specific DNA sequences associated with the Breast Cancer 1 (BRCA1) gene, as well as with the anticancer drug tamoxifen (TAM) and its associated DNA sequences. These interactions are analyzed using bare and multi-layered pencil graphite electrodes (PGEs). For the first time, carbon nanotube-modified screen-printed electrodes (SPEs) were utilized in this study. Biomolecular interactions between TAM and DNA were investigated using differential pulse voltammetry (DPV), which focused not only on the guanine signal but also on the oxidation reaction of TAM [82]. Research has developed a novel immunoassay that utilizes multiple polymer signal amplification strategies for the detection of the BRCA1 protein. This immunoassay is based on a large-surface glassy carbon electrode made from a composite material that includes silver nanoparticles (AgNPs) doped with poly(dopamine beta-cyclodextrin trimethylammonium bromide) (P[DAβCD/CTAB]) and functionalized mesoporous silica (MCM41SO3H). The constructed immunoassay platform allows for the effective immobilization of primary antibodies and provides excellent conductivity [83]. The reduced graphene oxide–yttrium nanocomposite (Y_2_O_3_–rGO/Apt/BSA) as a novel nanosensor was advanced for the detection of epidermal growth factor receptor (EGFR) antigen as a BC gene [84].

**Table 2:** Analytical and technical details of the advanced biosensors platform for ultra-sensitive detection of BRCA

Target	Technique	Platform	Matrix	Probe sequence	Linear range	LOD	Ref
BRCA-1	EC, EIS	SAM	Serum	5 COOH-GAT TTT CTT CCT TTT GTT C 3	1.0 fM to 10.0 pM	0.3 fM	[67]
BRCA-1	EC/DPV	SiO_2_@Ag/dsDNA/RhB	Real samples	SiO_2_@Ag/GCE	10 fM to 100 nM	2.53 fM	[68]
BRCA-1, 2	SPR	AuNPs/Graphene	Real samples	-	-	-	[69]
BRCA-1	EC/Colorimetric	-	Serum	-	50 pM to 200 nM	10 pM	[70]
BRCA-1	EC/CVs/DPV, EIS	ERGOh /PP3CAi modified GCE	Blood plasma	ERGOh/ GCE	10 fM–0.1 µM	3 fM	[71]
BRCA-1	EC/CVs/CA	Sandwich-type hybridization by capture probe		5 GAA CAA AAG GAA GAA AAT C 3	1 fM to 1 nM	1 fm	[72]
BRCA-1	EC/CVs	CNT/GC	Real samples	5 A-Biotin-IAC CTA ITC CTT CCA ACA IC		40 ng ml	[73]
BRCA-1	EC/DPV/HCR	Au electrode HCR	Human serum		1 aM to 10 pM	1 aM	[74]
BRCA-1	Optical	Optocoupler array	-	5 -TAAAACTAAATGTAAGAAAAAT-3	1 and 500 nM	0.9 nM	[75]
BRCA-1	Optical	Sandwich-type hybridization by capture probe immobilized on magnetic microparticles		5 GAGCATACATAGGGTTTCTCTTG GTTTCTTTGATTATAATTCATAC 3	200 to 800 nm	1 fM	[76]
BRCA-1	EC/EIS	Gene sequence based on lambda exonuclease assisted target recycling amplification	Clinical		0.1–10 nM	1 nM	[77]
BRCA-1	EC/EIS	SWCNT-SPEs	Real biological	5 -NH2-AGG-GTG-TCT-GAA-GGA-GGG-GG-3	5 and 160 g mL^− 1^	80 g mL^− 1^	[78]
BRCA-1	Optical/ (UV-vis)	AuNPs	-	-	1 fM to 1 nM	0.1 fM	[79]
BRCA-1	EC/DPV	ZnONWs/Au electrodes	Real samples	5’ AAT GGA TTT ATC TGC TCT TCG 3’	10.0 and 100.0 µM	3.32 µM	[80]
BRCA-1	EC/Optical	GO–DNA–FAM	-	FAM–CTTACCAGATGGGACAC	10 µg-400 µL	-	[81]
BRCA-1	EC/DPV	SPE/MWCNT	-	5’- GATTTT CTTCCT TTTGTT C-3’	2–10 µg/ml	-	[82]
BRCA-1	EC/DPV/SWV	P[DA-β-CD/CTAB])-AgNPs	Plasma		0.625–20 pg/mL	0.003 pg/mL	[83]
EGFR	FTIR, XRD, TEM, SEM	Y_2_O_3_–rGO/Apt/BSA	Serum		10 fg mL^–1^ to 100 ng mL^–1^	0.251 fg mL^–1^	[84]

## Conclusion and future prospective

Biosensor technology has emerged as one of the most researched areas due to its simplicity, speed, low cost, high sensitivity, and high selectivity, all of which contribute to the next generation of medical advancements. Consequently, the development of biosensors for medical and laboratory diagnostics is both attractive and important. Detecting biomarkers associated with various types of cancer is a top priority in biosensor development. In recent years, the advancement of biosensors related to breast cancer (BC) has become a key research focus for scientists. Recent progress in bioengineering and the use of fluorescently labeled nanomaterials have significantly enhanced the sensitivity limits of biosensors. Additionally, the incorporation of aptamers, nucleotides, affibodies, peptide arrays, and molecularly imprinted polymers provides innovative tools for developing improved biosensors compared to conventional methods.

Other advancements in biosensing technology include wearable sensors and artificial intelligence, which are aimed at precision medicine to enhance medical treatment. These innovations facilitate improved patient data collection and analysis by integrating biosensors with classical algorithms and pattern recognition techniques. The use of nanomaterials and nanotechnology has significantly increased the sensitivity of biosensors for in vitro diagnostics, while paper-based biosensor devices provide a cost-effective alternative that maintains high sensitivity and allows for sophisticated design implementation. According to the results obtained in this study, there appears to be a pathway to developing the ideal biosensor for gene detection. This biosensor can not only provide rapid and accurate results but also operate on a nanoscale. Such capabilities are crucial for both the early detection and monitoring of breast cancer.

## Data Availability

No datasets were generated or analysed during the current study.

## Abbreviations

BC: Breast cancer
EC: Electrochemical
SAM: Self-assembled monolayer
SPR: Surface plasmon resonance
BRCA: Breast Cancer gene
CV: Cyclic Voltammetry
DPV: Differential Pulse Voltammetry
EIS: Electrochemical Impedance Spectroscopy
PP3CA: PolyPyrrole-3- carboxylic acid
RGO: Reduced graphene oxide
CA: Chronoamperometry
CNT: Carbon-nanotubes
HCR: Hybridization chain reaction
PARP: Poly (ADP-ribose) polymerase
AR: Androgen receptor
CT: Computed tomography
LGR: Large genomic rearrangement
MMPs: Magnetic microparticles
UV-vis: Ultraviolet-visible
SPEs: Screen-printed carbon electrodes
PGE: Pencil graphite electrode
MWCNT: Multi-walled carbon nanotube
SSCP: Single-strand conformation polymorphism
MLPA: Multiplex ligation-dependent probe amplification
